# High-level mupirocin resistance in methicillin-resistant staphylococci isolated from dogs and cats

**DOI:** 10.1186/s12917-019-1973-y

**Published:** 2019-07-10

**Authors:** Magdalena Kizerwetter-Świda, Dorota Chrobak-Chmiel, Magdalena Rzewuska

**Affiliations:** Division of Microbiology, Department of Preclinical Sciences, Faculty of Veterinary Medicine, Warsaw University of Life Sciences, Ciszewskiego, 802-786 Warsaw, Poland

**Keywords:** *Staphylococcus aureus*, Staphylococcus pseudintermedius, Staphylococcus haemolyticus, ileS2

## Abstract

**Background:**

Mupirocin is one of the few antimicrobials active against methicillin-resistant *Staphylococcus aureus* (MRSA), and is frequently used for the eradication of MRSA nasal colonisation in humans. Initially, mupirocin resistance was recognised in human *S. aureus*, including MRSA isolates, then also among coagulase-negative staphylococci (CoNS). Nowadays, mupirocin resistance is occasionally observed in canine staphylococci, along with *Staphylococcus pseudintermedius* (MRSP) strains, as well as CoNS, which usually show methicillin resistance. In the current study, high-level mupirocin resistance in methicillin-resistant staphylococci isolated from diseased dogs and cats was investigated.

**Results:**

Among 140 methicillin-resistant staphylococci isolates from dogs and cats, three showed high-level mupirocin resistance in a screening test using the agar disk diffusion method. One was recognised as methicillin-resistant *S. aureus*, one as methicillin-resistant *S. pseudintermedius*, and one as methicillin-resistant *Staphylococcus haemolyticus*. *S. pseudintermedius* and *S. aureus* were isolated from dogs, *S. haemolyticus* was obtained from a cat. All isolates showed high-level mupirocin resistance, confirmed by minimum inhibitory concentration (MIC) values of above 1024 μg/ml and the presence of the plasmid-located gene *ileS2*. This is the first report on the detection of high-level mupirocin resistance (HLMR) in *S. haemolyticus* of feline origin.

**Conclusions:**

This study revealed the occurrence of HLMR in three *Staphylococcus* isolates obtained from companion animals in Poland. The results of this study indicate that the monitoring of mupirocin resistance in staphylococci of animal origin, especially in methicillin-resistant isolates, is strongly recommended.

**Electronic supplementary material:**

The online version of this article (10.1186/s12917-019-1973-y) contains supplementary material, which is available to authorized users.

## Background

Staphylococci are extremely versatile and constantly evolving microorganisms. One of the major concerns for public health is the spread of methicillin resistance within the *Staphylococcus* genus. Worryingly, methicillin-resistant strains typically also show multidrug resistance, in addition to resistance to virtually all β-lactam antibiotics [1–4]. Mupirocin is a topical antimicrobial used to treat superficial bacterial skin infections and to control the spread of methicillin-resistant *Staphylococcus aureus* (MRSA) in humans. Mupirocin resistance was at first recognised in *S. aureus* strains, and it is well characterised within this species. Two mupirocin-resistant phenotypes have been described: low-level and high-level resistance [5]. Low-level mupirocin resistance (LLMR) is attributed to a point mutation or mutations in the chromosomal *ileS* gene encoding isoleucyl-tRNA synthetase. Strains with such mutations are characterised by low mupirocin minimum inhibitory concentration (MIC) values falling within the range from ≥8 μg/ml to 256 μg/ml [6]. The mechanism of high-level mupirocin resistance (HLMR) is associated with a conjugative plasmid carrying the *ileS2* (*mupA*) gene encoding an additional isoleucyl-tRNA synthetase with reduced affinity for mupirocin. The MIC of mupirocin in the case of HLMR is ≥512 μg/ml [5]. Initially, mupirocin was used in human medicine, especially for the elimination of MRSA nasal colonisation and soon the increased prevalence of resistance was observed. After MRSA hospital outbreaks and frequent decolonisation in patients and the medical staff using mupirocin, resistance was even observed in up to 63% of locally isolated methicillin-resistant strains [7]. Moreover, mupirocin resistance was also found in coagulase-negative staphylococci (CoNS) [8].

In veterinary medicine, the increasing prevalence of infections caused by methicillin-resistant staphylococci is becoming a worrying issue. The most widespread methicillin-resistant species in dogs is *Staphylococcus pseudintermedius* (MRSP), but MRSA, as well as methicillin-resistant CoNS, are also isolated from companion animals [1–3, 9]. Generally, these strains show multidrug-resistance, significantly limiting the treatment options, and leading to the more frequent use of alternative antimicrobials, like mupirocin [4, 10–13]. Although mupirocin is only approved for use in animals in some countries, such as the United States [10], off-label use is a possibility for veterinarians. In Poland, mupirocin is not registered for use in animals. Nowadays, mupirocin resistance occasionally occurs in staphylococci isolated from companion animals [14–19]. However, it may be assumed, that these data are understated because susceptibility to mupirocin is not routinely determined for animal isolates. However, there is a lack of data regarding high-level mupirocin resistance in staphylococci of animal origin in Poland. The aim of this study was to investigate the prevalence and the mechanism of high-level mupirocin resistance among methicillin-resistant staphylococci isolated from diseased dogs and cats.

## Results

### Prevalence of HLMR among methicillin-resistant staphylococci

Of the 140 methicillin-resistant staphylococci isolated between 2007 and 2017, three (2.1%) isolates showed HLMR in a screening test using the agar disk diffusion method (Confidence interval, CI 95%: 0.7–6.1%) (Additional file 1: Figure S1). Two were obtained from dogs and one from a cat, in 2007, 2013, and 2016 respectively.

### Identification of high-level mupirocin-resistant staphylococcal isolates

Based on the results of standard bacteriological tests, all isolates were recognised as staphylococci, two were coagulase-positive, and one coagulase-negative. Using a *nuc*-specific PCR, one of the two coagulase-positive isolates was classified as *S. aureus* and the other as *S. pseudintermedius* (Additional file 1: Figure S2). The single coagulase-negative isolate was identified as *Staphylococcus haemolyticus* with APIStaph with a reliability of 82.2% and confirmed by sequence analysis of the 16S rRNA gene. According to the BLAST analysis, the sequence of the 16S rRNA gene displayed 100% concordance with a type strain of *S. haemolyticus* ATCC 29970 (GenBank: D83367.1), confirming the identification of our isolate as *S. haemolyticus*. The characteristics of the isolates are shown in Table 1.

**Table 1:** The characteristics of *Staphylococcus* isolates with HLMR used in the study

Isolate ID	Origin	Type of clinical sample	*mecA* presence	Identification
583/07	Dog	Bursa exudate	+	*S. aureus*/MRSA
813/13	Dog	Wound swab	+	*S. pseudintermedius*/MRSP
840/16	Cat	Abscess	+	*S. haemolyticus*/MRSH

### Antimicrobial susceptibility

All three isolates tested showed resistance to β-lactam antibiotics evaluated by the agar disk diffusion method: penicillin, amoxicillin, amoxicillin with clavulanic acid, ceftiofur, and oxacillin or cefoxitin according to the species of staphylococci tested. The *mecA* gene was detected in all isolates, confirming their methicillin resistance (Additional file 1: Figure S3). The results of the tests for antimicrobial susceptibility testing by agar disk diffusion method for non-β-lactam antibiotics using the agar disk diffusion method are presented in Table 2. All three isolates showed the multidrug- resistance phenotype with resistance to β-lactam antibiotics, fluoroquinolones, macrolides, lincosamides, and were high-level mupirocin resistant. Detailed resistance profiles for each strain are given in Table 2.

**Table 2:** The results of antimicrobial susceptibility testing to non-β-lactam antibiotics for studied staphylococcal isolates

Isolate ID/Species	Disk diffusion test		MIC (μg/ml) of mupirocin
	Resistant	Susceptible	
583/07 *S. aureus*	ENR, MAR, TET, ERY, L, RIF, MUP	GEN, SXT, CHL	> 1024
813/13 *S. pseudintermedius*	SXT, ENR, MAR, TET, ERY, GEN, L, CHL, MUP	RIF	> 1024
840/16 *S. haemolyticus*	SXT, ENR, MAR, ERY, GEN, L, MUP	TET, RIF, CHL	> 1024

SXT-sulfamethoxazole/trimethoprim, TET-tetracycline, GEN-gentamicin, ENR-enrofloxacin, MAR-marbofloxacin, ERY-erythromycin, CHL-chloramphenicol, L-lincomycin, RIF-rifampicin, MUP-mupirocin

### Mupirocin resistance

High-level mupirocin resistance was confirmed in all three staphylococcal isolates. The MIC values of mupirocin for the isolates were above 1024 μg/ml (Table 2). A specific *ileS2* gene fragment of 458 bp was detected in PCR for all isolates tested (Additional file 1: Figure S4). Comparison of the *ileS2* sequences of the three examined isolates revealed 100% identity with the previously published sequence of plasmidic *ileS2* gene from the mupirocin-resistant *S. pseudintermedius* strain HR547/11 (GeneBank: JX186508).

## Discussion

The three *Staphylococcus* isolates obtained from companion animals during routine bacteriological examination showed multidrug resistance, as well as methicillin and mupirocin resistance. Furthermore, the MIC value of mupirocin ≥1024 μg/ml and the presence of the *ileS2* gene confirmed HLMR in all isolates tested. The antimicrobial treatment history of the animals from which the isolates were obtained was not available; therefore, the impact of previous antibiotic use on the selection of the mupirocin-resistant resistant staphylococci cannot be assessed. Our results confirmed the occasional occurrence of high-level mupirocin resistance in staphylococci of animal origin reported previously by others. Recently, one MRSP isolate out of 110 *S. pseudintermedius* canine isolates tested in Korea showed HLMR [16]. Similarly, resistance to mupirocin was found in one out of 100 *S. pseudintermedius* isolates from healthy dogs in Australia, and this strain was also multidrug resistant [15]. In the USA, among 581 *S. pseudintermedius*, HLMR determined by the plasmidic *ileS2* gene was found in one methicillin-susceptible *Staphylococcus pseudintermedius* (MSSP) isolate [14]. Matanovic et al. described HLMR in one out of 102 *S. pseudintermedius* strains isolated from dogs in Croatia [17]. This strain was classified as MSSP, the *ileS2* gene was located on a conjugative plasmid, which additionally contained the aminoglycoside resistance *aacA-aphD* gene. In another study conducted in the USA, two mupirocin-resistant strains, one MRSP, and one methicillin-resistant *Staphylococcus sciuri*, were found among staphylococci isolated from dogs with superficial pyoderma. Studies conducted in England showed one canine MRSA in 204 examined *S. aureus* with a MIC of mupirocin 16 μg/ml, consistent with values assigned as low-level mupirocin resistance. In contrast to these results, in Canada all *S. pseudintermedius* isolates obtained from dogs with skin and soft tissue infections (*n* = 50) demonstrated susceptibility to mupirocin.

Antimicrobial resistance genes are easily transferred between staphylococci even among different species of the genus, which is particularly evident in the dissemination of the *mecA* gene [4]. Likewise, plasmid-mediated HLMR can disseminate horizontally and clonally. In vitro and in vivo transfer of the *ileS2* gene between *S. aureus* and *Staphylococcus epidermidis* was described [20]. Moreover, plasmids conferring the *ileS2* gene may also contain genes determining gentamicin, tetracycline or macrolide resistance. Exposure to any of these antimicrobials would coselect for the resistant strains. The three high-level mupirocin-resistant and methicillin-resistant isolates described in this study showed different resistance patterns to other antimicrobials; all were resistant to fluoroquinolones, macrolides, and lincosamides.

The emergence of mupirocin resistance among MRSA and other staphylococci in humans suggests that it may be potentially transferred to staphylococci of animal origin. The close contacts of owners with their dogs and cats favour the spread of resistant bacteria, including mupirocin-resistant ones. The transmission of mupirocin resistance genes from human *S. aureus* isolates to canine *S. pseudintermedius* is highly probable, although it may occur in both directions. However, selective pressure is the main factor contributing to the rise and increase in antimicrobial resistance. Recently, MRSP and other methicillin-resistant staphylococci have emerged as important pathogens in small animal veterinary medicine [1–3]. These bacteria typically show multidrug resistance, which significantly limits effective treatment options [11, 12, 21]. Topical mupirocin use in companion animals provides some therapeutic options for infections caused by methicillin-resistant strains, thus mupirocin use in veterinary practice may also increase in the coming years [13]. However, the use of this antimicrobial in veterinary medicine raises some concerns related to the idea that mupirocin should be reserved only for human medicine. Counteracting the increase in antimicrobial resistance including mupirocin can only be achieved through complex and coordinated actions, such as the One Health approach.

## Conclusions

In conclusion, this is the first report describing HLMR in multidrug-resistant and methicillin-resistant staphylococci isolated from companion animals in Poland. To our knowledge, this is also the first description of HLMR in *S. haemolyticus* of feline origin. Mupirocin resistance was previously found in *S. haemolyticus* strain isolates from humans and dogs [15, 22]. Imprudent antimicrobial use could cause HLMR strains to become more prevalent among animals in the future. The incidence of resistant bacteria is an important public health threat, thus surveillance of resistance, including mupirocin resistance among animal staphylococci, is strongly recommended. Moreover, the presence of HLMR among staphylococci obtained from the companion animals is of public health concern and emphasises the need for the introduction of antimicrobial stewardship programmes in veterinary settings.

## Methods

### Bacterial isolates

A collection of 140 methicillin-resistant staphylococci was investigated for the presence of high-level mupirocin resistance. All the isolates used in this study were obtained from clinical specimens of animal origin submitted to the Microbiological Diagnostic Laboratory, Faculty of Veterinary Medicine, Warsaw University of Life Sciences-SGGW in Poland during routine bacteriological examinations between 2007 and 2017. The types of clinical samples from which staphylococci were isolated are given in Table 1. The collection comprised of 126 canine and 14 feline isolates. The identification of staphylococci was based on standard bacteriological methods: colony morphology, Gram staining, catalase testing, coagulase production, and the slide agglutination test. Bacterial DNA was isolated using a DNA Genomic Mini kit (A&A Biotechnology) and lysostaphin (100 mg/ml) according to the manufacturer’s instructions. The amount and quality of DNA were determined using The Thermo Scientific NanoDrop™1000 Spectrophotometer. The identification of coagulase-positive staphylococci was confirmed by *nuc* gene PCR analysis [23]. The identification of the coagulase-negative isolate was based on the biochemical properties determined with APIStaph and confirmed by PCR amplification and sequence analysis of the 16S rRNA gene using universal primers [24]. The amplicon was sequenced using a 3730 xl DNA Analyzer. Sequencing files were analysed using the Chromas Lite version 2.33 program. Nucleotide BLAST analysis was carried out on the National Center for Biotechnology Information (NCBI) website (http://blast.ncbi.nlm.nih.gov).

### Antimicrobial susceptibility testing

Methicillin resistance was ascertained using the agar disk diffusion method with oxacillin (OXA-1 μg) or cefoxitin (FOX-30 μg) depending on the staphylococcal species [25]. Methicillin resistance was confirmed by *mecA* gene amplification by PCR as described by Strommenger et al. [26]. High-level mupirocin resistance was initially detected according to the method recommended by CLSI using 200 μg mupirocin disks [27]. Isolates showing no zone around the disk were regarded as high-level mupirocin-resistant and subjected to further testing. The 95% Confidence Interval was calculated using the Wilson score method [28]. The high-level mupirocin-resistant isolates were subjected to routine antimicrobial susceptibility testing using the agar disk diffusion method for a panel of the following antimicrobials: penicillin, amoxicillin, amoxicillin with clavulanic acid, ceftiofur, sulfamethoxazole/trimethoprim, tetracycline, gentamicin, enrofloxacin, marbofloxacin, and erythromycin. Extended susceptibility testing was performed for: mupirocin, chloramphenicol, lincomycin, and rifampicin. The CLSI veterinary guidelines [25] were used for interpretation of the agar disk diffusion testing results.

### Minimal inhibitory concentration of mupirocin

After the initial detection of HLMR using 200 μg mupirocin disk, the minimal inhibitory concentration of mupirocin was determined using the broth microdilution technique for 0.064–1024 μg/ml of mupirocin (Sigma) according to the CLSI guidelines [27].

### Detection of mupirocin resistance by PCR

The *ileS2* gene determining high-level mupirocin resistance was detected by PCR method described by Anthony et al. [29] using the primers ileS2F and ileS2R to amplify a 458 bp fragment of the *ileS2* gene.

### Sequence analysis

To confirm the PCR results, the obtained amplicons were sequenced using the primers forward and reverse. The sequences were compared with the *ileS2* sequences available in the Genbank using the nucleotide BLAST program (http://blast.ncbi.nlm.nih.gov).

## Additional file

Additional file 1: **Figure S1.** Disk diffusion method – detection of high-level mupirocin resistance using 200 μg mupirocin disk for methicillin-resistant *Staphylococcus pseudintermedius* isolate 813/13. **Figure S2.** Identification of *Staphylococcus aureus* (A) and *Staphylococcus pseudintermedius* (B) using *nuc*-specific PCR. Fig. S2A line 1: negative control, line 2: GeneRuler 100 bp Plus DNA Ladder (Thermo Scientific), line 3: 359 pb product obtained for *Staphylococcus aureus* isolate 583/07. Fig. S2B line 1: negative control, line 2: MassRuler DNA Ladder Mix (Thermo Scientific), line 3: 926 bp PCR product obtained for *Staphylococcus pseudintermedius* isolate 813/13. **Figure S3.** Identification of methicillin resistance in staphylococci - amplification of a 532 bp fragment of the *mecA* gene. Line 1: *Staphylococcus aureus* isolate 583/07, line 2: *Staphylococcus pseudintermedius* isolate 813/13, line 3: *Staphylococcus haemolyticus* isolate 840/16, line 4: MassRuler DNA Ladder Mix (Thermo Scientific), line 5: negative control. **Figure S4.** Identification of methicillin high-level mupirocin resistance in staphylococci - amplification of a 458 bp fragment of the *ileS2* gene. Line 1: *Staphylococcus aureus* isolate 583/07, line 2: *Staphylococcus pseudintermedius* isolate 813/13, line 3: *Staphylococcus haemolyticus* isolate 840/16, line 4: GeneRuler 50 bp DNA Ladder (Thermo Scientific), line 5: negative control. (DOCX 546 kb)

## Data Availability

All data obtained and analyzed in this study are included in this manuscript. The additional figures can be found in Additional file 1.

## Abbreviations

AMC: Amoxicillin with clavulanic acid
AMX: Amoxicillin
CHL: Chloramphenicol
CLSI: Clinical and Laboratory Standards Institute
CoNS: Coagulase-negative staphylococci
ENR: Enrofloxacin
ERY: Erythromycin
FOX: Cefoxitin
GEN: Gentamicin
HLMR: High-level mupirocin resistance
L: Lincomycin
MAR: Marbofloxacin
MIC: Minimum inhibitory concentration
MRSA: Methicillin-resistant *Staphylococcus aureus*
MRSH: Methicillin-resistant *Staphylococcus haemolyticus*
MRSP: Methicillin-resistant *Staphylococcus pseudintermedius*
MSSP: Methicillin-susceptible *Staphylococcus pseudintermedius*
MUP: Mupirocin
OXA: Oxacillin
PEN: Penicillin
RIF: Rifampicin
SXT: Sulfamethoxazole/trimethoprim
TET: Tetracycline
XNL: Ceftiofur
