# Supplementary material for: High-level mupirocin resistance in methicillin-resistant staphylococci isolated from dogs and cats
Source: BMC Vet Res. 2019 Jul 10;15:238. doi: 10.1186/s12917-019-1973-y (PMC6617863; doi:10.1186/s12917-019-1973-y)
Supplement: Supplementary file 1 — Figure S1. Disk diffusion method – detection of high-level mupirocin resistance using 200 μg mupirocin disk for methicillin-resistant Staphylococcus pseudintermedius isolate 813/13. Figure S2. Identification of Staphylococcus aureus (A) and Staphylococcus pseudintermedius (B) using nuc-specific PCR. Fig. S2A line 1: negative control, line 2: GeneRuler 100 bp Plus DNA Ladder (Thermo Scientific), line 3: 359 pb product obtained for Staphylococcus aureus isolate 583/07. Fig. S2B line 1: negative control, line 2: MassRuler DNA Ladder Mix (Thermo Scientific), line 3: 926 bp PCR product obtained for Staphylococcus pseudintermedius isolate 813/13. Figure S3. Identification of methicillin resistance in staphylococci - amplification of a 532 bp fragment of the mecA gene. Line 1: Staphylococcus aureus isolate 583/07, line 2: Staphylococcus pseudintermedius isolate 813/13, line 3: Staphylococcus haemolyticus isolate 840/16, line 4: MassRuler DNA Ladder Mix (Thermo Scientific), line 5: negative control. Figure S4. Identification of methicillin high-level mupirocin resistance in staphylococci - amplification of a 458 bp fragment of the ileS2 gene. Line 1: Staphylococcus aureus isolate 583/07, line 2: Staphylococcus pseudintermedius isolate 813/13, line 3: Staphylococcus haemolyticus isolate 840/16, line 4: GeneRuler 50 bp DNA Ladder (Thermo Scientific), line 5: negative control. (DOCX 546 kb) [file 12917_2019_1973_MOESM1_ESM.docx]

Additional file 1


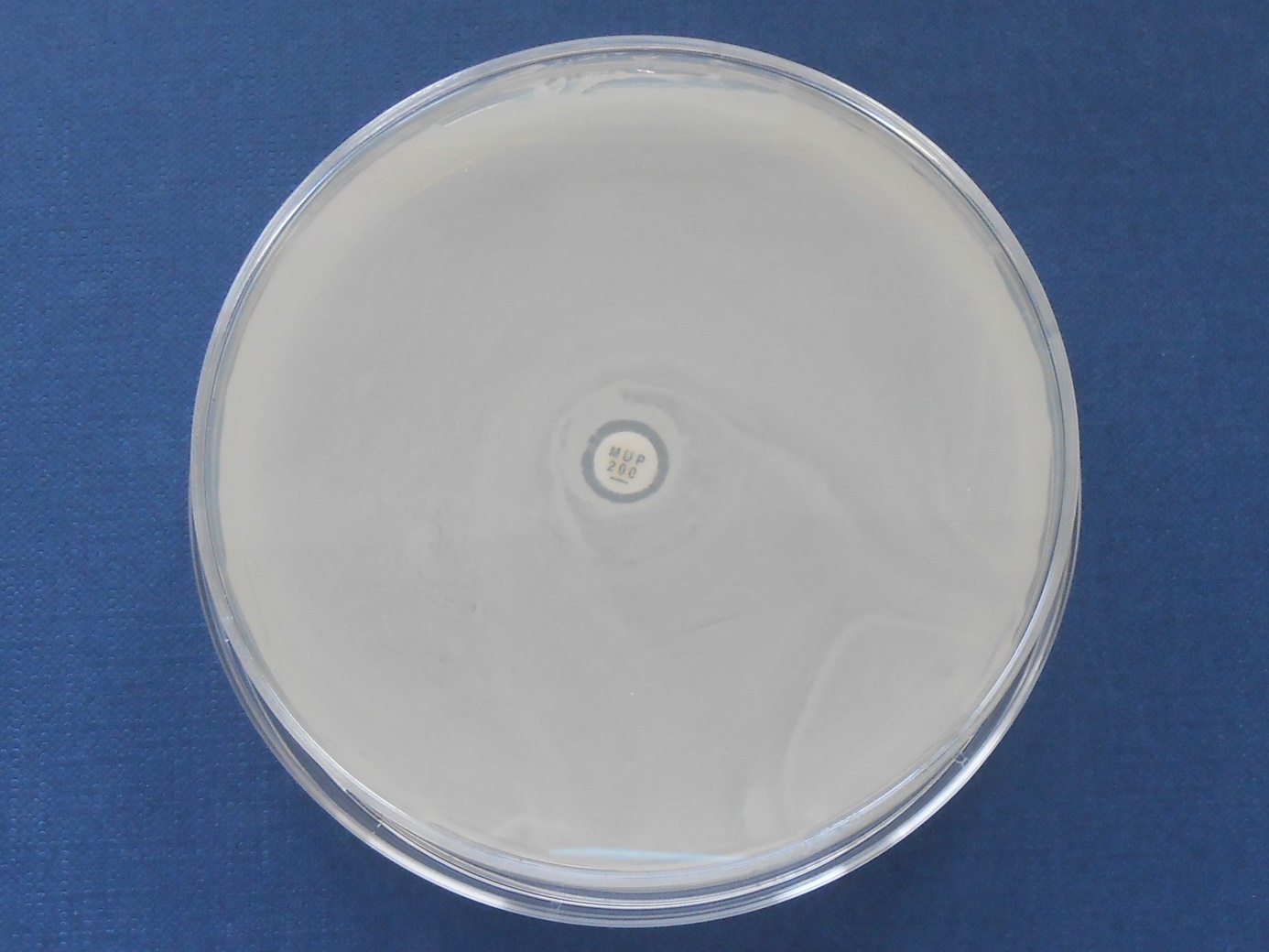


**Fig. S1** Disk diffusion method – detection of high-level mupirocin resistance using 200µg mupirocin disk for methicillin-resistant *Staphylococcus pseudintermedius* isolate 813/13.


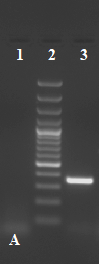

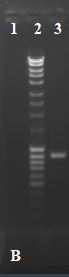


**Fig. S2** Identification of *Staphylococcus aureus* (A) and *Staphylococcus pseudintermedius* (B) using *nuc*-specific PCR. Fig. S2A line 1: negative control, line 2: GeneRuler 100 bp Plus DNA Ladder (Thermo Scientific), line 3: 359 pb product obtained for *Staphylococcus aureus* isolate 583/07. Fig. S2B line 1: negative control, line 2: MassRuler DNA Ladder Mix (Thermo Scientific), line 3: 926 bp PCR product obtained for *Staphylococcus pseudintermedius* isolate 813/13.


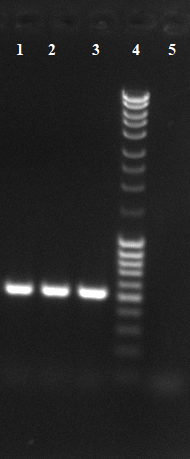


**Fig. S3** Identification of methicillin resistance in staphylococci - amplification of a 532 bp fragment of the *mecA* gene. Line 1: *Staphylococcus aureus* isolate 583/07, line 2: *Staphylococcus pseudintermedius* isolate 813/13, line 3: *Staphylococcus haemolyticus* isolate 840/16, line 4: MassRuler DNA Ladder Mix (Thermo Scientific), line 5: negative control.


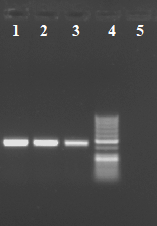


**Fig. S4** Identification of methicillin high-level mupirocin resistance in staphylococci - amplification of a 458 bp fragment of the *ileS2* gene. Line 1: *Staphylococcus aureus* isolate 583/07, line 2: *Staphylococcus pseudintermedius* isolate 813/13, line 3: *Staphylococcus haemolyticus* isolate 840/16, line 4: GeneRuler 50 bp DNA Ladder (Thermo Scientific), line 5: negative control.
